# The effect of a methyl group on structure and function: Serine vs. threonine glycosylation and phosphorylation

**DOI:** 10.3389/fmolb.2023.1117850

**Published:** 2023-02-10

**Authors:** Joseph J. Barchi, Caitlin N. Strain

**Affiliations:** Center for Cancer Research, Chemical Biology Laboratory, National Cancer Institute at Frederick, Frederick, MD, United States

**Keywords:** DNA methylation, serine, threonine, mucin-type glycosylation, GalNac O-linked glycans, phosphorylation, tumor-associated carbohydrate antigens (TACAs), conformation

## Abstract

A variety of glycan structures cover the surface of all cells and are involved in myriad biological processes, including but not limited to, cell adhesion and communication, protein quality control, signal transduction and metabolism, while also being intimately involved in innate and adaptive immune functions. Immune surveillance and responses to foreign carbohydrate antigens, such as capsular polysaccharides on bacteria and surface protein glycosylation of viruses, are the basis of microbial clearance, and most antimicrobial vaccines target these structures. In addition, aberrant glycans on tumors called Tumor-Associated Carbohydrate Antigens (TACAs) elicit immune responses to cancer, and TACAs have been used in the design of many antitumor vaccine constructs. A majority of mammalian TACAs are derived from what are referred to as mucin-type O-linked glycans on cell-surface proteins and are linked to the protein backbone through the hydroxyl group of either serine or threonine residues. A small group of structural studies that have compared mono- and oligosaccharides attached to each of these residues have shown that there are distinct differences in conformational preferences assumed by glycans attached to either “unmethylated” serine or *ß*-methylated threonine. This suggests that the linkage point of antigenic glycans will affect their presentation to the immune system as well as to various carbohydrate binding molecules (e.g., lectins). This short review, followed by our hypothesis, will examine this possibility and extend the concept to the presentation of glycans on surfaces and in assay systems where recognition of glycans by proteins and other binding partners can be defined by different attachment points that allow for a range of conformational presentations**.**

## Introduction

Since the beginning of the 21st century, discoveries in the biomedical and “chemical-biological” sciences have outpaced even what those in these fields could have imagined. 1) Unraveling the human genome, 2) the realization that RNA has arguably surpassed DNA in its number of critical cellular functions and 3) the approval of game-changing tumor immunotherapeutic agents "Three examples of the many discoveries that have advanced our understanding of human diseases in the past 20 years are: 1). The tools we now have to decipher increasingly complex biological problems will continue to help scientists and physicians alleviate suffering and death from disease with greater efficiency and selectivity.

As we continue to tackle the harder to answer questions and focus on the more minute details of disease pathology, there are certain, features of the discovery process that all investigators must continually consider. What one feature could these seemingly disparate discoveries have in common? Possibly put another way, what feature is common in the unraveling of any biological/biochemical/cellular discovery that involves nucleic acids, proteins, lipids or carbohydrates? Without question, this feature is the relationship of molecular *Structure* with biological *Function*. In organic chemistry, one of the first things we learn are the details of molecular structure and how molecules are arranged in 3-dimensional space. When organic chemistry meets biology (as well as many other disciplines including, but not limited to, physics and materials science), that is where the “Function” feature becomes important, and thus the sub-field of Structure-Function Relationships emerges. In medicinal chemistry, the traditional acronym used was SAR, for Structure-Activity-Relationships. In the early days of compound screening and medicinal chemistry, changes to small molecule structures that led to altered activities were interpreted mostly on intuition about “potential” intermolecular interactions in the absence of structural information on a molecular level. The advent of X-ray crystallography, advanced NMR techniques and now Cryo Electron Microscopy (CryoEM) have allowed actual visualization of molecular interactions and expanded interpretations of SAR to the atomic level. Computational approaches (Chai et al., 2021; Diwan et al., 2021) to structure have also advanced dramatically and can complement experimental techniques or even, at times, at times completely substitute for hard structural data. (Diwan et al., 2021; Zhou et al., 2022). With the information available today, interpretation of function cannot adequately be predicted if the structures of interacting molecules are unknown. Only a small fraction of the picture will emerge without a 3-dimensional rendition of the system under study.

**FIGURE 1:**
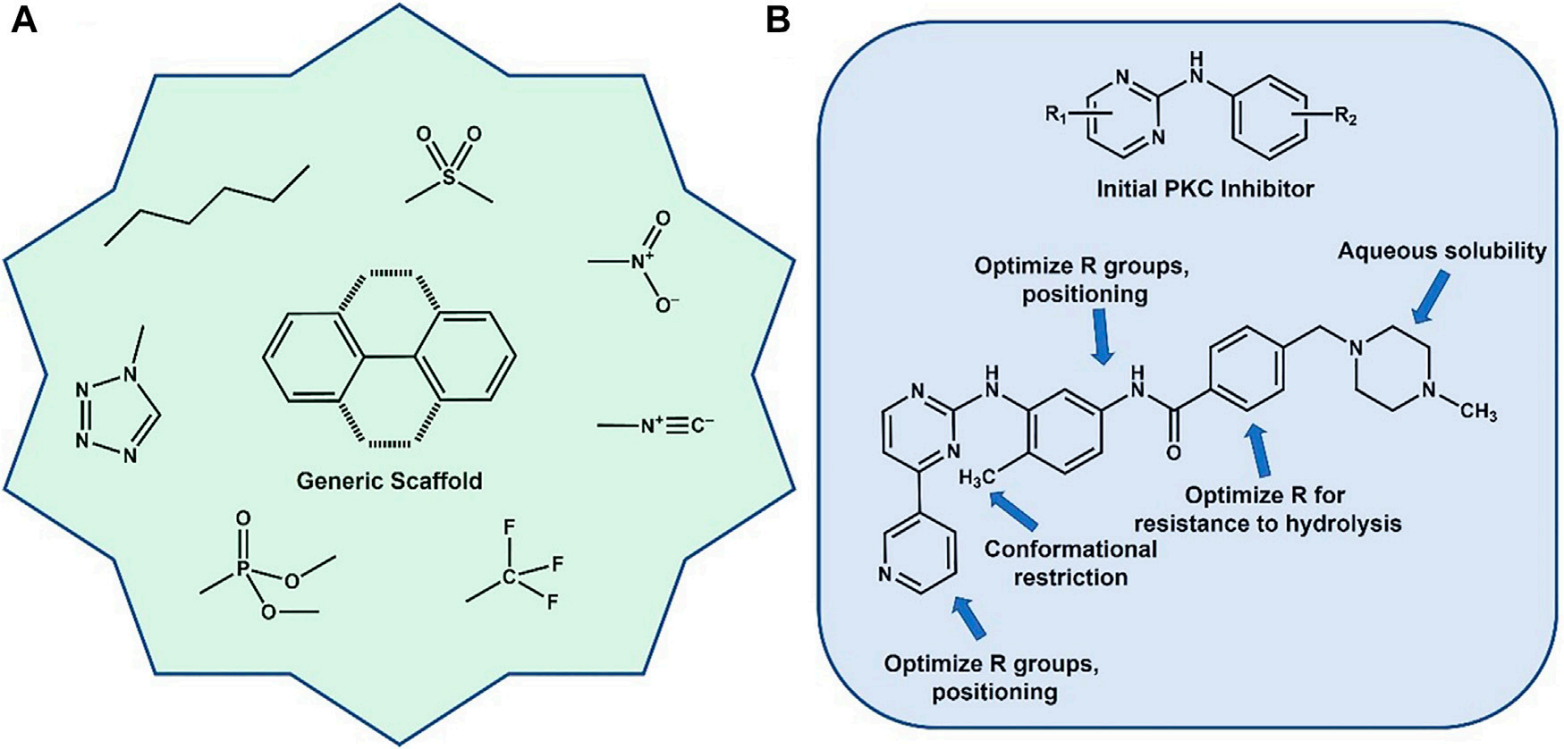
**(A)** Generic aromatic scaffold surrounded by typical functional groups used in various medicinal chemistry campaigns. **(B)** Evolution of the development of Gleevec (Imatinib) for CML. Optimization included addition of a critical methyl group to help lock in a specific preferred conformation.

The determination of both qualitative and *quantitative* SAR (Cronin et al., 2003; Gedeck and Lewis, 2008; Bak, 2021) allows the unraveling of the structural changes, both on the molecular and atomic levels, that alter biological activity and often cause large adjustments in function. Some of the typical “Med Chem” adjustments (Guha, 2011; Hoffer et al., 2018; Broccatelli et al., 2019; Das et al., 2022; de Esch et al., 2022; Klein et al., 2022) made in a lead optimization campaign are shown in Figure 1A. It is now well known that often simple and what may look like relatively minor changes in structure can cause dramatic modulations in biological activity. Regarding this review, the simple structural change that will be discussed is the presence or absence of a methyl group (*vide infra*). An impactful example of the effect this simple group can exert on a drug discovery campaign is the design of Imatinib (Gleevec) considered the first “targeted therapy” (Figre 1B). Gleevec inhibits the B*cr-Abl* kinase that drives Chronic Myelogenous Leukemia (CML) cell proliferation, essentially shutting down the disease state. A short schematic of the evolution of the final drug molecule is shown where functional groups “grew” out of a pyrimidine-anilino scaffold, (identified as a Protein Kinase C (PKC) inhibitor) with various fragments occupying key areas of the protein kinase binding pocket. Relevant to the subject of this review is the addition of an *ortho*-aromatic methyl group on the diaminobenzyl ring. This led to a restriction of rotational freedom of the surrounding rings, leading to greater specificity for the *Bcr-Abl* kinase over PKC. (Avendano and Menendez, 2015). This is but one (very successful) example of where a methyl group made a substantial contribution to the design of a billion dollar drug that will potentially save millions of lives. (Jonsson and Wilking, 2007)

While this is an example of a beneficial effect of drug methylation, there are a host of reasons for using methyl groups in medicinal chemistry. An excellent review by Barreiro, et al., outlines the many uses of methylation in the design of active agents for clinical use. (Barreiro et al., 2011). The following list are some of the more relevant uses of methylation in medicinal chemistry:

1) Increased lipophilicity causing lower solubility and improved membrane penetration
2) Electron donating inductive effects leading to differential receptor binding
3) Participation in CH-π interactions (in aromatic agents and nucleic acid packing)
4) Induction of folding though hydrophobic interactions after methyl group introduction
5) Inductive effects that modulate tautomerization (see the history of cimetidine) (Parsons and Ganellin, 2006)

### CH_3_ and gene expression

Early work in genetics suggested that we each have a unique set of genes that defines all our physical and emotional traits: hair color, skeletal density and pigmentation, as well as our aptitude for math vs. language or even propensities to acquire certain diseases. (Yan and Zhou, 2004; Zhang, 2020; Suzuki, 2022). We now know that epigenetics—a second layer of genetic instructions—can dictate when and whether certain genes are turned on or off. And when one thinks of epigenetics, more than likely the first process that comes to mind is *methylation,* in particular, DNA methylation. This is a process that can modulate the expression of genes by affecting *major* structural rearrangements *via*, what could be considered, a very *minor* structural change: the addition of four atoms (CH_3_). When the DNA wrapped in the nucleosomes of chromatin structures is unmethylated, the structures are “relaxed” allowing binding of transcription factor proteins and initiation of gene expression. Methylation of cytidine residues along with modifications in histone “tails” both facilitate a tightening of the DNA wound around histone proteins, preventing access to their backbones effectively turning off gene expression.

How does the small methyl group do this? Although high resolution structural information is lacking, there are many structural studies using various biophysical techniques that have narrowed the consequences to several possibilities (Li et al., 2022a; Li et al., 2022b): 1) Adjustment of the BI-BII rotamer ratio about the 3′-phosphate bond that leads to steric clash with the adjacent 5′-sugar residue, 2) Non-productive methyl group interaction during protein-DNA binding (more specifically, in transcription factor/DNA binding) leading to modulation of transcription, and 3) a global adjustment to the 3-dimensional structures of nucleosomes and chromatin. The aforementioned steric clash allows more flexibility to the DNA backbone and disallows certain H-bonding interactions, thus in turn affecting chromatin structure and dynamics. While these features have been accepted, there is still debate as to the actual structural consequences of DNA methylation.

**FIGURE 2:**
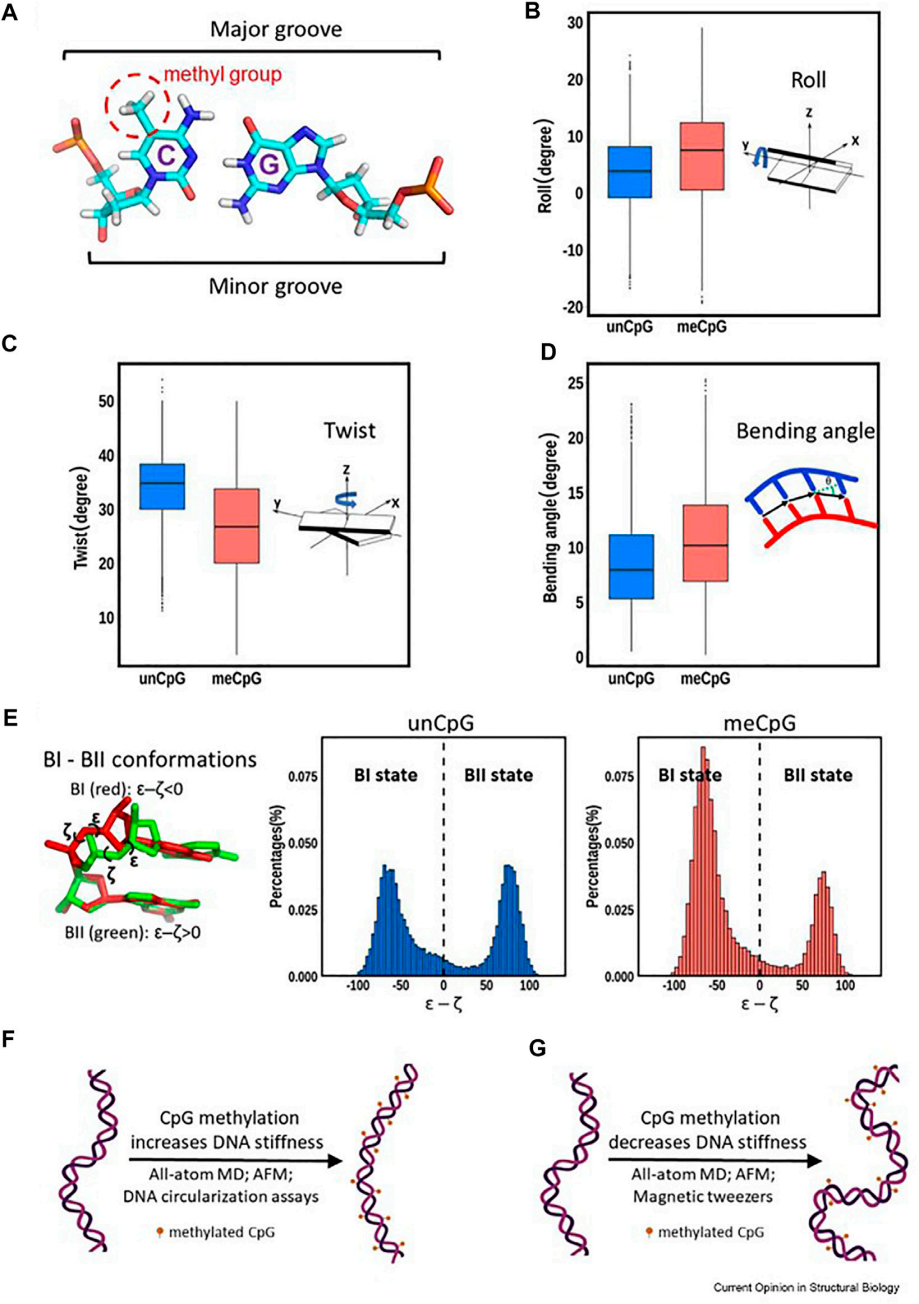
Various structural changes from DNA methylation (reused from open access reference, Li, et al. (Li et al., 2022a). Panel **(A)** depicts the 5′-cytidine methyl group points toward the major groove. Panels **(B–D)** illustrate the differences in roll, twist and bending angle between unmethylated and methylated DNA. Panel **(E)** shows a movement toward the BI state of DNA upon methylation. Panels **(F)** (Perez et al., 2012) and **(G)** (Shon et al., 2019) show that conflicting data regarding the effect of methylation on DNA dynamics have been reported depending on the techniques used.

Conflicting data has been generated among the different biophysical techniques used to measure DNA stiffness/flexibility and how these changes in methylated DNA modulate nucleosome structures and hence drive function (Figure 2F and citations (Portella et al., 2013; Yeou et al., 2022). It is generally accepted that certain canonical DNA structural features, such as Twist and Roll, are affected more strongly through methylation. (Carvalho et al., 2014). The cytosine 5-methyl group points into the major groove providing added hydrophobicity to the standard DNA base pairing. Figure 2 summarizes some of these findings.

***Histone Methylation***. In addition to methylation by DNA methyltransferases that add CH_3_ to the five position of cytosine bases, the proteins that make up the nucleosome, known as histones (of which histones 1, 2 three and four are known) are also post-translationally modified with methyl groups. (Jenuwein, 2006). These modifications primarily occur on lysine amino groups, where a total of three methyl groups can be added, creating a newly quaternary nitrogen atom with a net positive charge. They can also occur on arginine residues, but far less often than lysine. The function of this modification is similar to DNA methylation and the two work in concert: Histone methylation can cause gene expression and/or repression depending on the status of the specific methylation patterns and dynamics of the methyl transfer (from methyltransferases) and demethylation (caused by demethylases). (Kako et al., 2019). There are a host of these epigenetic enzymes and associated proteins that are involved in these processes. There is a coordinated interplay between the various participants which are commonly called “writers”, “readers” and “erasers”. Writers such as DNA methyltransferases (DNMTs), histone acetyltransferases (HATs), and histone lysine methyltransferases (HMTs) transfer a specific group (e.g., a methyl or acetyl group) to either DNA or histones. Erasers such as histone deacetylases (HDACs) and histone lysine demethylases (KDMs) remove these groups and readers such as bromodomains and extra-terminal binding proteins (BETs) or methyl-histone binding proteins (MBDs) recognize and bind these modified domains. (Javaid and Choi, 2017; Li and Li, 2021). Inhibitors of each of these protein families have been devevloped as anticancer agents since many are overexpressed in various tumors. (Zhong et al., 2021).

### The methyl group on amino acids

It is obvious that a group that may be considered structurally “inconsequential” can induce a variety of critical changes in the functioning of a cell, and for that matter, the organism in which the changes are taking place. Epigenetic changes are the most “consequential” adjustments that can be imparted to a genome through the function of simple methyl groups. Several amino acids that contain methyl groups are, in their own right, important for protein function, folding and conformational adjustments/preferences. There are six amino acids that contain methyl groups (alanine, leucine, isoleucine, valine, threonine and methionine). Among other functions, these contribute to protein hydrophobicity, thus being involved in formation of hydrophobic pockets during protein folding. Interesting work has been performed that have specifically “isolated” certain methyl groups in various aspects of protein structure and function. Methyl groups have been deemed protein plasticizers; i. e, they add to flexibility and distendability, increasing the dynamics in regions of high methyl content, which in turn can affect, for example, the mobility of enzyme catalytic sites. (Nickels et al., 2012). Methyl groups can contribute to packing in transmembrane domains of small proteins called traptamers, where single groups can modulate protein-protein interactions. (He et al., 2017).

**FIGURE 3:**
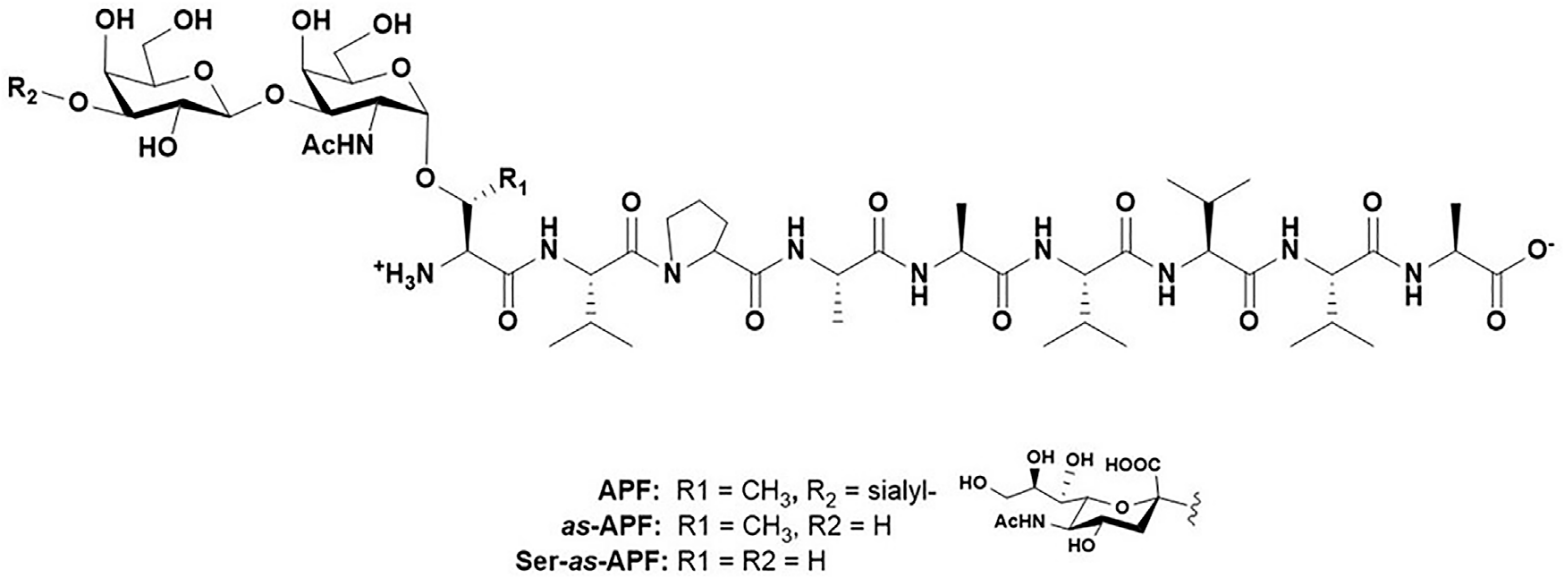
Structure of Antiproliferative factor (APF), *as*-APF and Ser-*as*-APF.

For the purposes of this review, the focus will be the one methyl group on threonine residues in peptides, proteins and glycopeptides. Several studies (outlined below) have shown that when comparing serine to threonine (hereafter referred to as Ser and Thr, respectively), the acceptor specificity of enzymes that modify the hydroxyl groups of these residues, as well as the resulting structural composition of these post-translational modified entities (specifically, glycosylation and phosphorylation), can be distinctly different. Our interest in this stemmed from the observation we made several years ago on an interesting glycopeptide isolated from the urine of patients with a disease called Interstitial Cystitis/Painful Bladder Syndrome (IC/PBS). IC/PBS is characterized by a thinning of the bladder epithelium down to a few cell layers with a concomitant decrease in bladder-coating glycosaminoglycan structures, leading to urinary urgency, frequency and severe pain. (Dasgupta and Tincello, 2009; Marcu et al., 2018). A discovery in 1996 by the Keay lab strongly suggested that this, at the time, unknown substance halted proliferation of normal bladder epithelial cells in these patients, and hence may have been a distinct causative agent of IC/PBS. (Keay et al., 1996). Careful structural characterization and synthesis prove the molecule to be the simple glycopeptide shown in Figure 3 called Antiproliferative factor (APF). (Keay et al., 2004). This structure and its de-sialylated analogue were found to have equal antiproliferative potency. However, when the glycosylated Thr residue was changed to a Ser during comprehensive structure activity studies, the antiproliferative potency dropped four orders of magnitude. (Kaczmarek et al., 2008).

Comprehensive conformational analysis by NMR and molecular modeling revealed distinct structural differences between the serine and Thr APF: The natural Thr-containing glycopeptide populated a folded state where the glycan rotated over the first three amino acids, whereas the serine derivative was more flexible and extended. (Mallajosyula et al., 2013). Similar differences had already been shown with other Ser/Thr glycopeptide pairs: A review written by one of us (JJB) in 2013 illustrated that in every instance where fully characterized structures were compared for Ser/Thr mucin-type O-linked glycopeptide pairs, the Thr-conjugated glycan was always less flexible and populated a much more restricted conformational space than that sampled by a Ser-linked O-glycopeptide. (Barchi, 2013). Our research and several other works published in the 2000s have solidified the concept of differential reactivity and functions of Ser-versus Thr-linked O-glycopeptides.

### Serine vs. threonine O-linked glycosylation: Origins of selectivity

Mucin-type O-linked glycosylation is a unique form of protein post-translational modification (PTM) that differs distinctly from prominent N-linked glycosylation: While N-linked glycans are produced by the *en bloc* transfer of a large oligosaccharide to a specific amino acid sequon (the asparagine sidechain in NXS/T motifs, where X = any amino acid but proline), followed by post synthetic trimming and adjustments, mucin-type O-linked glycans are synthesized by “one-at-a-time” addition of individual monosaccharides, starting with N-acetyl galactosamine (GalNAc). Initiation of O-linked glycan synthesis is done by a series of enzymes called UDP-GalNAc: polypeptide N-acetylgalactosaminyltransferases (GALNTs). There are now 20 isoforms of this enzyme which can be divided into distinct subgroups based on genomic structure, sequence homology and substrate specificities. Of the known isoforms of GALNTs, many of their structures (Fritz et al., 2004; de las Rivas et al., 2018; Fernandez et al., 2019) and acceptor specificities (Yang et al., 1992; Oconnell et al., 1992; Elhammer et al., 1993; Chou et al., 1995; Nehrke et al., 1997; Yoshida et al., 1997; Gerken et al., 1997; Elhammer et al., 1999; Gerken et al., 2006; Gerken et al., 2008; Kong et al., 2015; Revoredo et al., 2016; de las Rivas et al., 2017; Mohl et al., 2020; Coelho et al., 2022) have been studied by a wide range of research groups. Many of these seminal studies date back to the late 1970’s. (Young et al., 1979). While individual enzymes have their own idiosyncrasies, some general rules for acceptor substrate preferences have been determined, primarily by *in vitro* work using various peptide families. (Kong et al., 2015; Revoredo et al., 2016; de las Rivas et al., 2017; Bennett et al., 2012; Daniel et al., 2020; Rivas et al., 2018; Gerken et al., 2013). The proteins themselves have a common catalytic domain akin to other glycosyltransferases, but are unique in that they also contain a ricin-like lectin domain attached to the catalytic unit by a short 15–25 amino acid linker. (Gerken et al., 2013; de las Rivas et al., 2019). There is no common amino-acid sequon that directs mucin-type O-linked glycosylation, such as what is known for N-linked glycosylation on asparagine residues, but the aforementioned studies have identified several amino acid “preferences” surrounding a Ser or Thr glycosylation site. For example, there seems to be a distinct PXP motif preference, with Proline residues positioned at the +1 and +3 amino acids positions to the C-terminal side of an acceptor Thr or Ser site, where X represents a hydrophobic amino acid (the “proline pocket”). (Yoshida et al., 1997). N-terminal to the acceptor Ser/Thr also shows a preference for proline at either the -3 or -1 position with various hydrophobic residue preferred at position -2 (see Figure 1 in reference (de las Rivas et al., 2019)). The presence of the lectin domain serves to direct additional glycosylation by binding to an existing O-linked glycan site, while a new, as-yet-to-be-glycosylated residue, distant from the bound lectin domain is further glycosylated by the catalytic domain. This is primarily seen in mucin-type sequences where highly dense O-linked glycans often exist in clusters. Clearly, the regulation of mucin-type glycosylation is highly complex process where the interplay of various transferases, relevant peptide sequences and existing glycopeptides motifs coordinate to construct defined glycosylation patterns. (Coelho et al., 2022; Konstantinidi et al., 2022).

As may be expected, a good number of these studies have concentrated on the Ser vs. Thr specificity and the molecular basis behind some of this apparent selectivity. (Oconnell et al., 1992; Yang et al., 1992; Oconnell and Tabak, 1993; Wang et al., 1993; Muller et al., 1997; Gerken et al., 2006; Gerken et al., 2008; Perrine et al., 2009; Daniel et al., 2020). In GalNAc-T2, for example, Ser vs. Thr specificity seems to also be controlled by the presence of a pocket that accommodates the Thr methyl group and may explain the preference why GalNAc-Ts may favor glycosylating a Thr over a Ser residue. (de las Rivas et al., 2019; Fritz et al., 2006; Lira-Navarrete et al., 2015).

To date, several reports have suggested that acceptor preferences were different for either Ser or Thr residues. While it is difficult to pinpoint a specific publication where this discovery was first made, a series of studies in the 1990s began to unravel the acceptor specificity of various GALNTs using libraries of different peptide sequences that contained amino acids in motifs, mimicking proteins that had several known O-glycan sites. (Oconnell et al., 1992; Yang et al., 1992; Elhammer et al., 1993; Oconnell and Tabak, 1993; Wang et al., 1993; Gooley and Williams, 1994; Pisano et al., 1994; Chou et al., 1995; Gerken et al., 1997; Muller et al., 1997; Nehrke et al., 1997; Yoshida et al., 1997; Elhammer et al., 1999). Not surprisingly, many of these sequences incorporated both Ser and Thr residues that were present in either random assemblies, or where two otherwise identical sequences were compared by mutating one (Ser/Thr) residue for another. Thus, both deliberately and randomly, glycosylation of Ser and Thr residues have been compared regarding rates of reaction and glycosylation efficiency.

One of the first studies that compared Ser to Thr directly was by the Tabak group at in 1993. (Oconnell and Tabak, 1993). Here they studied the ability of extracts from rat salivary glands, kidneys and liver (as sources of GALNT’s) to glycosylate peptide sequences derived from von Willibrand factor (VWF) and erythropoietin (EPO) containing a residue known to be glycosylated *in vivo*. They found that in a natural peptide sequence from VWF containing a known glycan acceptor Thr residue, the extracts were virtually inactive against a Ser-mutant of this peptide sequence. Similarly, an EPO sequence with a known Ser acceptor site was glycosylated 20-fold less than a Thr mutant, even though this was the “unnatural” sequence. Crude structural data from Circular Dichroism (CD) spectra suggested a more conformationally defined disposition for the Thr-containing sequences compared to a more random coil conformation in the Ser-containing motifs. They reported that previous studies also showed that some transferases were determined to be [sic]“threonine-specific”. (Yang et al., 1992; Wang et al., 1993).

### O-linked threonine vs. serine glycosylation: 3-Dimensonal conformation

**FIGURE 4:**
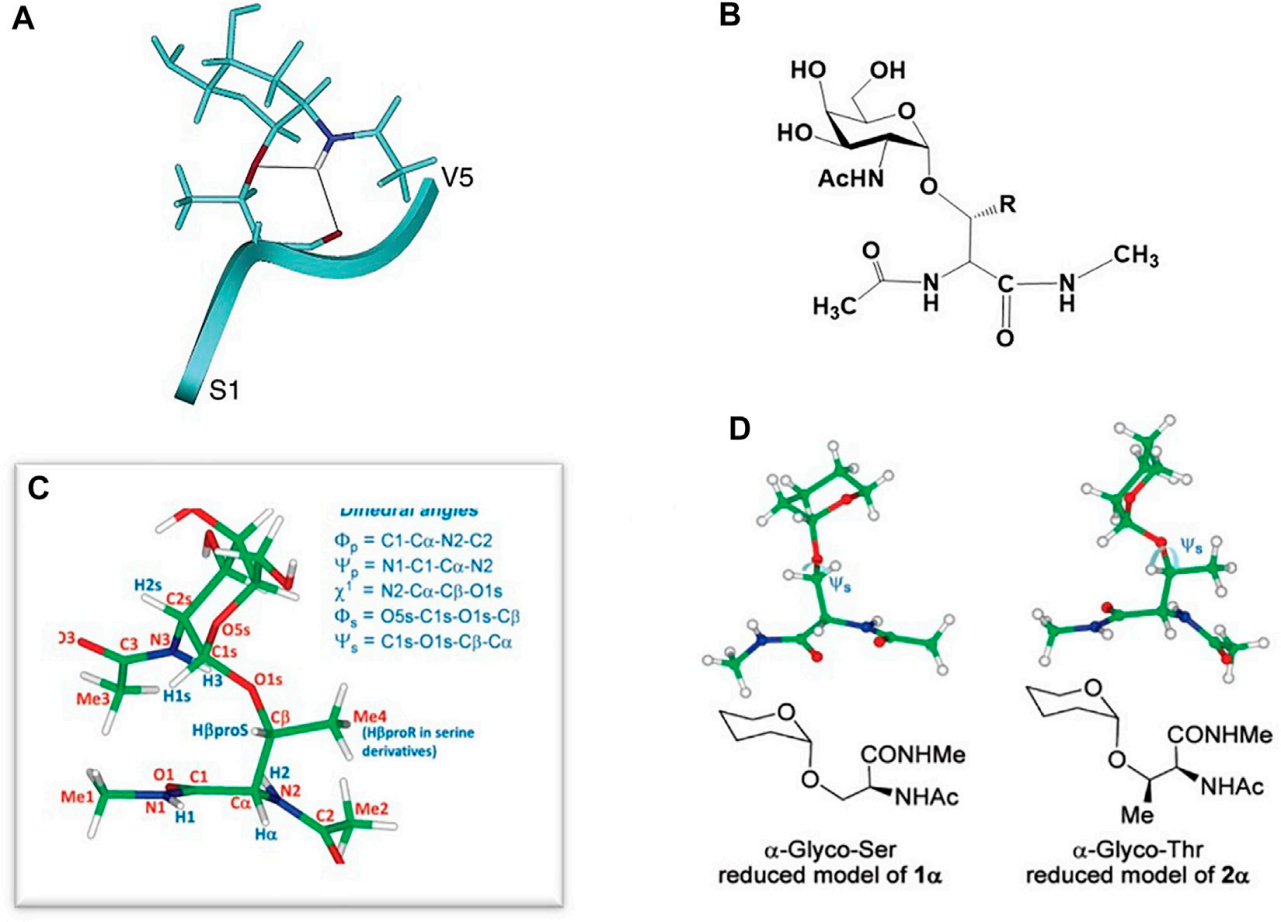
**(A)** Peptide-GalNAc interactions of one of the penta-glycopeptides studied in (Coltart et al., 2002). **(B)** Structure of the small glycopeptide studied in (Corzana et al., 2007). **(C)** Definition of the dihedral angles of the molecule in **(B)**. **(D)** Disposition of the sugar relative to the amino acid backbone in the Ser (left) and Thr (right) models of the molecule in **(B)**.

The work described above on GALNT Ser/Thr enzyme substrate specificity motivated those in the field to attempt to structurally define any differences that may exist between these two glycoamino acid substructures. As I pointed out in our review on NMR structures of mucin-type glycopeptides ((Barchi, 2013), mostly all work that compared conformational preferences of Ser vs. Thr-linked O-glycopeptides (either deliberately or randomly) showed that the Thr-linked glycopeptide motifs were conformationally more limited than those attached to Ser. Our group was one of the first to show this in a larger glycopeptide derived from the HIV-1 V3 loop. (Huang et al., 1997). Calculated structures of 24 residues glycopeptides showed that a Thr-linked GalNAc residue sampled a more limited conformational space as that of a Ser-linked GalNAc in the same molecule. Two years later, a similar study by Levine and coworkers on the structure of glycopeptides from MUC7 showed identical behavior of Ser and Thr-linked glycoamino acid motifs when they were present in the same glycopeptide. (Naganagowda et al., 1999). A comprehensive study of small, clustered O-linked glycopeptides in this area comes from Live, et al., who determined the 3-dimensional structures of several (penta)-glycopeptides with a contiguous Ser-Thr-Thr motif glycosylated with either the tumor-associated antigens Tn (GalNAc), TF (Gal-β1,3-GalNAc) or 2,6-sialyl TF (Gal-β1,3-(α-2,6-Neu5Ac)GalNAc) glycosylation. (Coltart et al., 2002). Interestingly, they found that many of the NMR and structural features of each system were similar, independent of the glycan attached. The conformational preferences were dictated by an interaction of the α-GalNAc unit with the peptide backbone. Extension to higher sugars did not affect this preference. The affect was absent in molecules where the glycan was attached *via* a *ß*-linkage to the Ser/Thr residue; this places the sugar in an extended position which does not allow easy interaction with the peptide backbone through hydrogen bonds. Molecular dynamics calculations showed that the small peptides were in an extended *ß*-strand-type configuration and highly organized around the glycosylated residues (Figure 4A). In all these NMR studies, a consistent theme was that the organization surrounding the α-O-GalNAc residue was dictated by hydrogen bonding that was found between the GalNAc NHAc unit and the backbone of the Thr residue, usually to the carbonyl oxygen. This hydrogen bond was not widely observed for the Ser-containing glycoamino acids residues. Moderately strong Nuclear Overhauser Enhancements (NOE’s) were sometimes observed between the NHAc methyl group and the peptide backbone.

In 2006, the group of Corzana and coworkers began to carefully and comprehensively compare Ser vs. Thr O-linked glycans on a molecular level by NMR and molecular modeling. (Corzana et al., 2007; Corzana et al., 2009; Bermejo et al., 2018). Their first report on a simple GalNAc-Ser conjugate suggested the interaction between the sugar and the peptide backbone was not a driver of a particular 3-dimensional conformational preference but bridging water molecules between the sugar and peptide were responsible for holding a specific conformation in place. (Corzana et al., 2006). These results (mildly) contradicted the aforementioned work of Live and others. A subsequent study showed that in very small glycopeptide models (Figure 4B) there were distinct differences between GalNAc-O-linked to either Ser or Thr at the dihedral angle connecting the amino acid *ß*-carbon with the glycosidic oxygen atom (Psi angle Ψs, Figure 4C). (Corzana et al., 2007) The origin of this effect was determined to be facilitated by two discoveries: 1) The difference in the way each molecule organizes a water shell surrounding the sugar and peptide and 2) The repulsion between the endocyclic sugar oxygen and the *ß*-methyl group in the Thr derivative which adjusts the aforementioned Psi dihedral angle to (stable) ∼120 deg; in the Ser analogue the angle is “anti” at 180 deg and considerably more flexible (Figure 4D). This study was performed on the “simplest” of glycopeptides, i.e., a single Thr or Ser amino acid that was capped at the N- and C-terminus with a small amide linkage (Figure 4B). This could be considered a shortcoming of the work as a longer peptide chain will have a more distinct conformational preference that may lead to more non-bonded interaction between the carbohydrate and the peptide backbone. However, this was arguably the first paper to describe the O-GalNAc-Thr/Ser differences and assign the preferred sugar-amino acid dihedral angles on an atomic level.

Other works (see (Barchi, 2013) and references therein) have shown similar results when comparing the NMR characterization the Tn antigen attached to Ser or Thr. The studies initially suggested and eventually proved that there is a distinct difference between the disposition a GalNAc orients itself when α-O-linked to either a Ser or Thr. The question remained: Does this structural difference dictate unique biological recognition and subsequent function? According to our own limited experience with APF the answer is a definite yes. But can we determine if a specific function can be attributed to these different structures? Is the biology of an organism modulated by this simple difference in glycoamino acid presentation? Several areas of research suggest that, similar to DNA methylation, relatively major biological and/or cellular changes can be attributed to whether a post translational modification is attached to a Ser or Thr.

### Recognition and functional consequences of Ser vs. Thr PTMs

Limited past and more comprehensive recent work, beginning with the above discussion, has started to unravel the unique conformational properties in Ser vs. Thr glycopeptides and the effect these may have on their function. A well-known example is the structure activity relationships of antifreeze glycoproteins (AFGP). These proteins are found in arctic fish and help allow these and other organisms to survive in supercooled water by their unique binding to ice crystals, resulting in a separation of water freezing and melting temperatures (hysteresis). AFGPs consist of a repeating tripeptide sequence -Ala-Ala-Thr-where the Thr residue is glycosylated with the aforementioned TF antigen. SAR of this motif determined that 1) both the glycan and the anomeric stereochemistry were essential for activity, and 2) substitution of the Thr residue for Ser also eliminated the hysteresis effect. This is a prime example of a complete ‘switch” in biological activity between Thr and Ser-linked O-glycopeptides. (Tachibana et al., 2004). Interestingly, many analogues of the tri-glycopeptide repeat, including many with Carbon-linked (C-Linked) carbohydrates, have been synthesized where the antifreeze properties are maintained. (Eniade et al., 2003; Capicciotti et al., 2011; Leclere et al., 2011).

**FIGURE 5:**
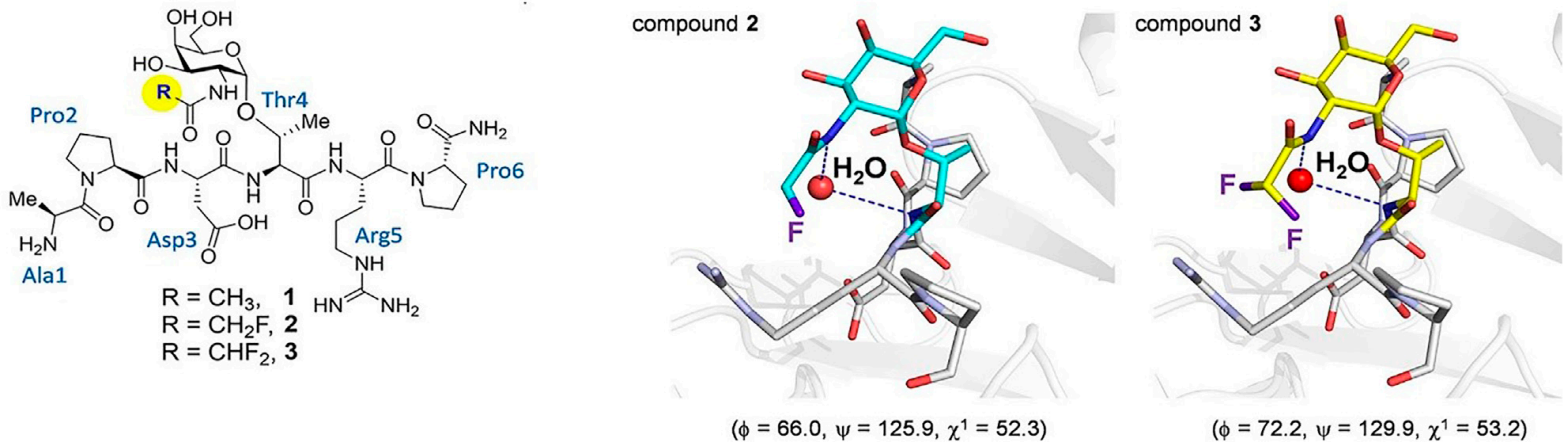
On the left, the three glycopeptides used to determine the presence of bridging water in the conformation of Tn-Thr taken from (Bermejo et al., 2018). On the right shows views of the binding sites of the complexes between glycopeptides two and three and the SM3 antibody (PDB IDs: 6FZR and 6FZQ, respectively), showing the key water molecule. The geometry of the glycosidic linkage is shown in parentheses and these angles closely match those calculated in solution.

In the past decade, many reports have further corroborated the importance of the Thr methyl group in biological recognition and activity. In 2009, Corzana and coworkers extended their structural analysis to dipeptides with a saccharide on either a Ser or Thr in Ser-Thr dipeptide pairs and saw similar organization in the Thr glycoamino acids when comparing side-by-side glycosylated sites. (Corzana et al., 2009). Since these Ser-Thr groupings are often present in mucin peptide repeats that are used in anticancer vaccine design, the conclusion was that the conformational preference will be important in the interaction of theses motifs with the human immune system. This postulate was borne out in subsequent studies that show specific interaction of MUC1 glycopeptides with antibodies that are raised to these antigens. (Martinez-Saez et al., 2015). SM3 is a monoclonal (mAb) antibody raised to a mucin from skim milk that had been stripped of many of its covalently attached glycans. (Burchell et al., 1987). SM3 has been shown to bind to MUC1 glycopeptides containing a GalNAc in the principle immunodominant sequence of Pro-Asp-Thr*-Arg (where the asterisk represents glycosylation with GalNAc). Using synthetic peptides with either the wild type Thr or a Ser substitution, (where both were either glycosylated or “naked”), an intriguing study showed that the nature of the GalNAc determinant is important for antibody recognition. The Thr-linked GalNAc tandem repeat peptide binds SM3 much more strongly than the Ser-linked analogue in Biolayer Interferometry (BLI) experiments. Smaller models of these glycopeptides were used to solve crystals structures of both the Thr and Ser-linked Tn structures. It was found that the Ser analogue adopts a high energy conformation about the glycosidic linkage in the crystal structure that is populated about 20% free in solution. These phi/psi angles are disallowed by the presence of the *ß*-methyl group in the Thr analogue. It was concluded that the nature of the aglycone dictates the organization of conformation around the glycosylation site. In 2018, a follow up to this work set out to prove that the bridging water molecules in the simulations described in the previous section were actually responsible for the different conformational preferences in Tn-Thr vs. Tn-Ser residues. (Bermejo et al., 2018). This was shown by solving the crystal structure of SM3 bound to a small Tn-Thr glycopeptide model that contained an N-monofluoro or difluoroacetyl groups on the GalNAc nitrogen in an effort to enhance potential interatomic hydrogen bonds that could mediate a bridging water structure. The results did show that in the fluorinated molecules, the bridging water molecule could be visualized for the first time (Figure 5.)

In a separate study, Mazal, et al., showed that another Tn-selective antibody showed distinct binding preferences for clustered Tn-containing glycopeptides depending on whether those clusters were comprised of Tn-Ser or Tn-Thr. (Mazal et al., 2013). Eight mAbs were raised to clusters of either S*S*S*, S*T*T* or T*T*T*. For example, the S*S*S*-specific mAb was unreactive toward tri-peptide clusters that contained Tn-Thr. In addition, this mAb did not bind tumor cells, whereas the ones raised to clusters with Thr residues did bind. While the MUC1 sequence does not contain any tri-Ser repeats, other mucins overexpressed on tumor cells do. These results could mean that the Tn-GalNAc presentation is critical for cellular recognition and ability to elicit an antitumor therapeutic effect from anti-Tn mAbs; this presentation is dependent on the underlying amino acid sequence.

This selective recognition capacity was also extended to plant lectins, proteins that are often used to determine the presence or absence of a particular glycosylation pattern on various cell types. (Dan et al., 2016). Another study, again by the Corzana group, determined that the binding of certain plant lectins have a distinct preference for either the Thr or Ser conjugated Tn antigen. (Madariaga et al., 2014). Three lectins that recognize O-linked GalNAc were studied: Soybean agglutinin (SBA), Vicia villosa agglutinin (VVA) and Helix pomatia agglutinin (HPA). All of these Carbohydrate Binding Proteins (CBPs) have been used to determine the presence of GalNAc-containing glycans on various cancer cells. (Panda et al., 2014; Silva and Rangel, 2017; Parameswaran et al., 2018). Interestingly, SBA and VVA strongly prefer a glycopeptide with a Thr-linked GalNAc whereas HPA prefers a Ser-linked sugar. Solution structures of Thr- and Ser-linked smaller glycopeptide models recapitulated the structures that were determined in the authors’ previous studies; they quote: “The different conformational behavior of the two Tn biological entities, the residues of the studied glycopeptides in the close proximity to the Tn antigen and the topology of the binding site of the lectins are at the origin of these differences.” This result confirms that there is a fine specificity of interactions of carbohydrate recognition proteins with glycoamino acids of these two aglycones.

### Other Ser and Thr hydroxyl group post-translational modifications

Ser and Thr residues are also sites for the ubiquitous and well-studied PTM, phosphorylation, which is a biological switch that is well known to turn mitogenic signaling (and a host of other cellular processes) on and off. They are also glycosylated with other sugars (Mannose, glucose, fucose and xylose) along with the intriguing and biologically critical *ß*-N-acetylglucosamine GlcNAc modification (O-GlcNAc) (for a comprehensive review of this and all aspects of glycobiology, see (Varki et al., 2022)). While glycosylation obviously affects the conformation and recognition capacity of O-linked glycopeptide motifs, are there similar changes associated with two of the most disease-related PTMs: Phosphorylation and O-GlcNAc-ylation?

**FIGURE 6:**
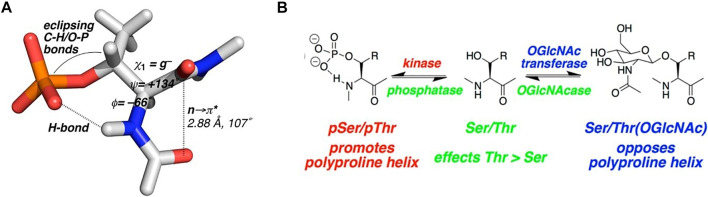
**(A)** Proposed phosphothreonine structure from NMR data of model tau peptides. **(B)** Results summary of the structural studies of model tau peptides with both phosphate and O-GlcNAc modifications (derived from reference (Brister et al., 2014)).

Work by Zondlo and co-workers showed some interesting findings regarding the effects of phosphorylation and O-GlcNAc modifications in model peptides. Their work initially concentrated on tau, a natively disordered protein that, when functioning normally is a component of microtubule scaffolding. Hyperphosphorylation of tau, however, promotes aggregation and formation of neurofibrillary tangles in many neurodegenerative diseases, including Alzheimer’s disease. Thus, tau is one of the entities responsible for the pathology of these disease. Since the pathological effects of tau are only evident after it is post-translationally modified, much of this group’s work concentrated on defining the effect of the phosphate (or GlcNAc) on the structure and conformation of both the modified Ser or Thr residue, and the surrounding sequence, in a series of model peptides from the tubulin-binding domain of tau. Phosphorylation of this domain is responsible for the aggregative and fibril-forming properties of the protein. Their first study showed that phosphorylation of peptides derived from the proline rich domain of tau nucleate the formation of a polyproline-II type helical structure in these sequences. (Bielska and Zondlo, 2006). While many previous studies showed phosphorylation-induced structural changes in peptides, many of those results showed more of an ordered-to-disordered transition, e.g., loss of helical content upon modification. (Johnson and Lewis, 2001; Andrew et al., 2002). A follow up study in 2014 looked more deeply into the effects of PTMs on Ser/Thr residues where they defined a specific “structure” of a phosphothreonine residue compared to that of a phosphoserine residue. (Brister et al., 2014). In this study, they showed a particular “conformational order” induced by a phosphothreonine that was not the same as a phosphoserine. Specifically, phosphothreonine residues, on average, were responsible for much greater structural adjustments in the model peptides than phosphoserine residues. This was observed mostly through changes in NMR data (coupling constants, induction of chemical shift changes, and amide H-bonding/temperature coefficients). Like other effects of mucin-type glycosylation, phosphorylated threonine residues were more structured and caused more conformational restriction than phosphoserine residues. Figure 6 shows the proposed structure of the phosphothreonine residue as determined by NMR. O-GlcNAc and phosphorylation seemed to have opposing roles, with phosphorylation, but not GlcNAcylation, promoting a PPII helix structure. The fact that many Ser/Thr sites compete for phosphorylation and O-GlcNAc modifications suggests that phosphate-induced conformational changes that lead to neurofibrillary tangles and the pathological effects in neurodegenerative disease are nullified with the presence of O-GlcNAc at those same sites. Inhibitors of the O-Glycanase that removes O-GlcNAc from Ser/Thr residues are being tested as therapeutic agents against neurodegenerative diseases. (Yuzwa et al., 2008; Yu et al., 2012; Yuzwa et al., 2012; Wang et al., 2020; Alteen et al., 2021).

Another study from this group also showed that in model alpha-helical peptides, both phosphorylation and O-GlcNac modifications stabilized the helix, but the effect was again more pronounced for phosphothreonine modifications. (Elbaum and Zondlo, 2014). This is further confirmation that the *ß*-methyl group of Thr can impart distinct differences to the structure and function of proteins/peptides when comparing PTMs on Ser or Thr residues.

## Discussion

Nature uses a variety of ways to chemically dictate the physical instructions necessary for differential cellular functions; these ultimately lead to the construction of intact organisms with specific traits. Structural chemists can now examine these at an atomic level and define the minute changes that funnel each instruction in a particular direction. The macromolecules that make up our cells—proteins, nucleic acids, carbohydrates and lipids—contain structural units that function in a variety of ways to drive 3-dimensional folding, conformation, assembly and binding interactions. Often, very minute changes in structure can redirect, or even reverse specific interactions. Functional groups such as those shown in Figure 1 can cause stereo-electronic, steric, dipole and non-bonding effects that modulate function in important biological settings. If researchers who discover, design and develop therapeutics agents have information that dictates *what* group may affect *which* function, there is a higher chance of success in any drug discovery campaign. This review briefly discussed the effect that a simple methyl group can have on structure and function, and there are many examples of this. The question posed here can be summarized: Are the function and recognition of well-known, post-translational modifications, in particular O-glycosylation, different for those linked to the γ-methyl group-containing Thr amino acid residue relative to those linked to a Ser that lacks this γ-methyl group? From the data that is available at present, it can be stated unequivocally that there are differences in the conformation and hence the presentation of glycans linked to a Ser hydroxyl group vs. a Thr hydroxyl group. As we have seen above, this can also translate to recognition and function, although the relevant number of reports is still quite limited. However, from the discussions presented here and the handful of studies that show distinct differences in biological recognition between mucin type glycans attached to Ser or Thr, some postulates and a general hypothesis can be proposed:

1) The solution and bound conformations of Tn-Ser vs. Tn-Thr are, up to now, always distinct from one another which suggests a “selection” process for protein binding
2) The conformational adjustments afforded by the methyl group should extend to binding of other higher order saccharides linked by mucin-type glycosylation

**Hypothesis:** Nature uses the Thr methyl group to “fine tune” either “promiscuity” or “specificity” into molecular recognition of mucin type glycoproteins/glycopeptides.

**FIGURE 7:**
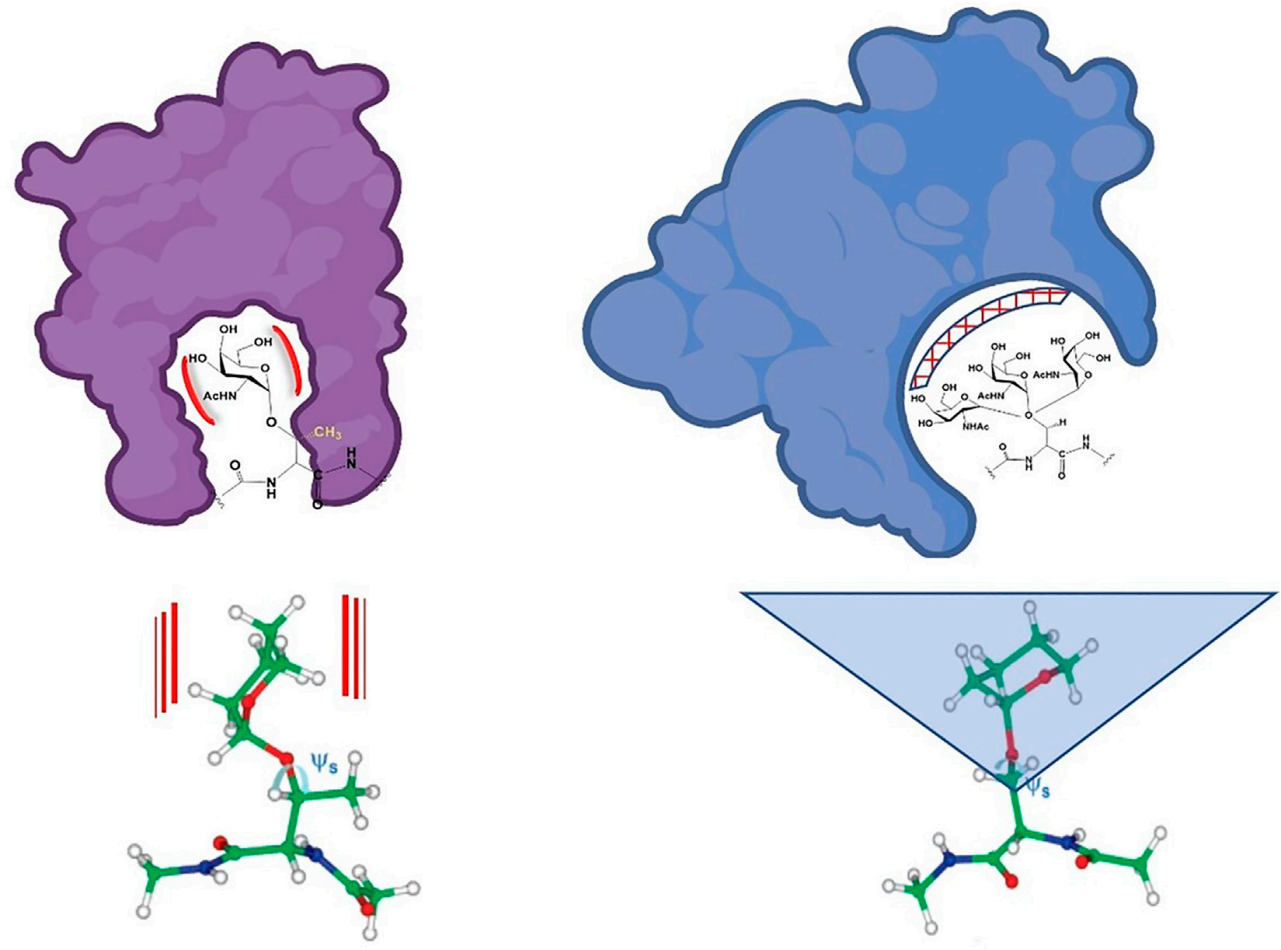
Schematic approximation of potentially “natural” binding modes of Ser and Thr-linked O-glycopeptides. The limited conformational space swept by the Thr analogues are shown on the left and the more flexible Ser conformational space is shown on the right. The calculated structures from reference (Gerken et al., 2013) are shown below. Red lines and blue triangle represent rigidity (Thr glycoamino acid) and flexibility (Ser glycoamino acid).

This is depicted in Figure 7. The limited range of angle “swept” out by the Thr glycoaminoacid adds “specificity” to a binding interaction whereas the more flexible Ser glycoaminoacid can bind more “promiscuously”, perhaps within different paralogs of CBPs. Said differently, if a more selective process is warranted, nature glycosylates and directs binding to O-linked Thr. If nature warrants a structure to be recognized by a series of say, closely related and biologically relevant lectins, it directs the interaction toward a O-linked Ser. This would have consequences with regard to processes such as cell adhesion and immune responses.

Similar to other work cited within this review, our lab is interested in developing anticancer vaccines based on mucin type glycopeptide structures. A recurrent theme and contention in this field is that the antigen that will elicit the optimum response is one that most closely resembles, not the actual gross structure that a tumor cell biosynthesizes on the cell surface, but the *presentation* of that epitope: i.e., the conformation that our immune system actually *sees*. That presentation may not be mimicked by a synthetic antigen that is now removed from its intracellular environment. This is the reason why a TACA along with the peptide to which it is covalently attached are both essential to elicit a proper immune response. (Trabbic et al., 2021). While this is still not a mimic of the cell surface, the platform on which the vaccine is constructed can help with this design to more closely resemble the cell and hopefully aid in proper presentation, for example, with regard to multivalency. Therefore, the Thr amino acid may aid in this presentation by restricting the epitope to (hopefully) the correct presentation. A very recent study by the Corzana group reported on the preparation of various vaccines made up of the MUC1/Tn antigen epitope and showed that conformational restriction using a Thr or other unnatural amino acids and/or sugar analogues that maintained a restricted presentation elicited higher antibody titers and bound more efficiently to tumor cells that are known to display that structure. (Asin et al., 2022). This would be considered precisely what was described above: Certain structures must mimic the cell surface presentation much better than others.

The conclusions that may be reached by the work presented in this short review help to validate the concept that the simple change from a “non-methylated” to “methylated” amino acid can change the profile of presentation, binding properties and immune recognition of that particular glycopeptide motif. Additional work in ours and other laboratories will hopefully further solidify this postulate and aid in the development of therapeutically useful agents in the future.

## Glossary

SAR: Structure-Activity-Relationship
CryoEM: Cryo Electron Microscopy
NMR: Nuclear Magnetic Resonance
CML: Chronic Myelogenous Leukemia
PKC: Protein Kinase C
CH3: Methyl group
Ser: Serine
Thr: Threonine
IC/PBS: Interstitial cystisis/painful bladder syndrome
APF: Antiproliferative factor
PTM: Post-translational modification
GALNT: Polypeptide N-actylgalactosaminyltransferase
EPO: Erythropoietin
VWF: Von Willibrand factor
CD: Circular Dichroism
TF: Gal-β1,3-GalNAc/Thomsen-Freidenreich antigen
Tn: GalNac
NOE: Nuclear Overhauser Enhancement
AFGP: Antifreeze glycoproteins
MUC1: Mucin protein one
SM3: Mucin antibody raised from skim milk
PDB: Protein Databank
mAb: Monoclonal antibody
SBA: Soybean agglutinin
VVA: Vicia villosa agglutinin
HPA: Helix pomatia agglutinin
CBP: Carbohydrate binding protein
DNMT: DNA-methyl transferase
HAT: histone-acetyl transferase
HMT: histone-methyl transferase
HDAcs: histone deacetylation protein
KDM: lysine demethylase
BET: bromodomains and extra terminal binding proteins
MBD: methyl-histone binding protein
BLI: biolayer interferometry
